# Time‐Restricted Feeding Prevents Cardiac Aging by Entraining Gut Microbiota Clock and Promoting Diurnal Rhythm Autophagy in Prediabetic Mice

**DOI:** 10.1002/mco2.70910

**Published:** 2026-08-09

**Authors:** Yuying Zhou, Zhiyi Yin, Xunxi Deng, Hejun Jiang, Ying Zhou, Yongjun Wang, Liyao Fu, Shi Tai

**Affiliations:** ^1^ Department of Cardiology The Second Xiangya Hospital of Central South University Changsha China; ^2^ Department of Blood Transfusion The Second Xiangya Hospital of Central South University Changsha China

**Keywords:** autophagy, cardiac aging, diurnal rhythms, diabetes, time‐restricted feeding

## Abstract

Time‐restricted feeding (TRF) is a promising dietary strategy for delaying metabolic aging, yet its efficacy in cardioprotection across metabolic stages remains poorly understood. This study demonstrates that a 10‐h TRF window significantly mitigates cardiac aging phenotypes including fibrosis, mitochondrial dysfunction, and senescence‐associated secretory phenotype in prediabetic mice, whereas these protective effects are largely blunted at the diabetic stage, despite improving systemic metabolism in both prediabetic and diabetic models. Mechanistically, TRF acts as a chronotherapeutic cue that restores the diurnal oscillations of short‐chain fatty acid (SCFA)‐producing gut microbiota. These rhythmic SCFAs serve as peripheral “metabolic zeitgebers” that resynchronize cardiac diurnal rhythms autophagy. In vitro, SCFAs enhance autophagic flux and preserve mitochondrial integrity in senescent cardiomyocytes. Crucially, the cardioprotective benefits of TRF are largely abolished in p62‐deficient mice, identifying p62‐dependent autophagy and mitochondrial homeostasis as a key molecular mechanism underlying TRF efficacy. In overt diabetes, the loss of TRF‐mediated protection is attributed to a profound “metabolic rigidity” that prevents restoration of autophagic rhythmicity. Overall, our findings reveal that TRF prevents cardiac aging by entraining the gut microbiota–SCFA–cardiac p62 axis during the prediabetic stage, highlighting a critical metabolic window for dietary intervention.

## Graphical abstract

The graphical abstract image presents cardiac and intestinal changes in prediabetic and diabetic mice after time restricted feeding.

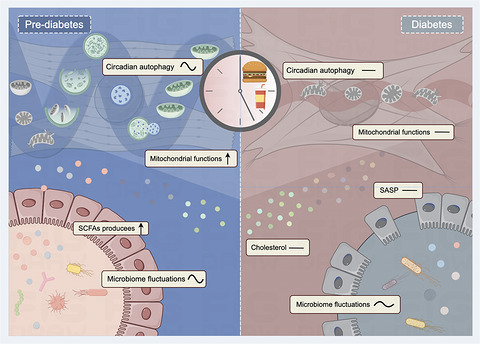

## 1 Introduction

Cardiac aging is a complex pathological process that serves as a primary driver for heart failure and various cardiovascular complications [1, 2]. This process is drastically accelerated under diabetic conditions [3], where chronic metabolic stress and systemic inflammation precipitate premature senescence in cardiomyocytes, leading to interstitial fibrosis, pathological hypertrophy, and impaired compliance. While senescent cells have emerged as key therapeutic targets, the search for nonpharmacological interventions that can effectively delay or reverse this “metabolic aging” remains a major challenge in cardiovascular medicine [4].

Time‐restricted feeding (TRF), a dietary regimen that consolidates caloric intake into a consistent daily window, has shown remarkable potential in extending health span and ameliorating metabolic syndrome in preclinical models [5, 6, 7, 8]. However, its clinical translation has been hindered by puzzlingly inconsistent outcomes [7, 9, 10, 11, 12]. While some trials report significant cardiometabolic improvements, others show neutral or even paradoxical effects, particularly concerning cardiovascular mortality in patients with established metabolic diseases. We hypothesized that this inconsistency stems from a fundamental but overlooked variable: the stage‐dependent efficacy of TRF relative to the progression of gluco‐metabolic dysregulation. Specifically, the transition from prediabetes to overt diabetes is characterized by an escalating “metabolic rigidity [13],” which may fundamentally alter how the heart perceives and responds to rhythmic feeding cues.

The heart's resilience depends heavily on the synchronization of its peripheral circadian clock with systemic metabolic signals [14]. Autophagy, the primary cellular clearance mechanism for damaged organelles, is a key executor of this rhythmic protection. Recent evidence suggests that TRF can entrain circadian autophagy [15, 16, 17, 18, 19], yet whether this entrainment can bypass the profound metabolic desynchrony inherent in advanced diabetes remains unknown. Furthermore, emerging research highlights the gut–heart axis as a vital conduit for interorgan communication. Gut‐derived metabolites, particularly short‐chain fatty acids (SCFAs), act as “metabolic zeitgebers” that could potentially synchronize cardiac autophagy with the feeding cycle [20, 21, 22].

In this study, we aimed to bridge these knowledge gaps by rigorously comparing the efficacy of TRF in attenuating cardiac aging in prediabetic versus overtly diabetic mouse models. We focused on the gut microbiota–SCFA–cardiac p62 axis as the central mechanism. Our findings reveal that the cardioprotective benefits of TRF are remarkably potent in the prediabetic stage but are largely abolished once diabetes is established. This study provides a critical mechanistic framework for the clinical application of TRF, emphasizing that the timing of intervention and the preservation of metabolic plasticity are essential for successful cardiac protection against metabolic aging.

## 2 Results

### 2.1 TRF Ameliorates Metabolic Dysfunction and Reduces Adiposity in Prediabetic Mice

**FIGURE 1:**
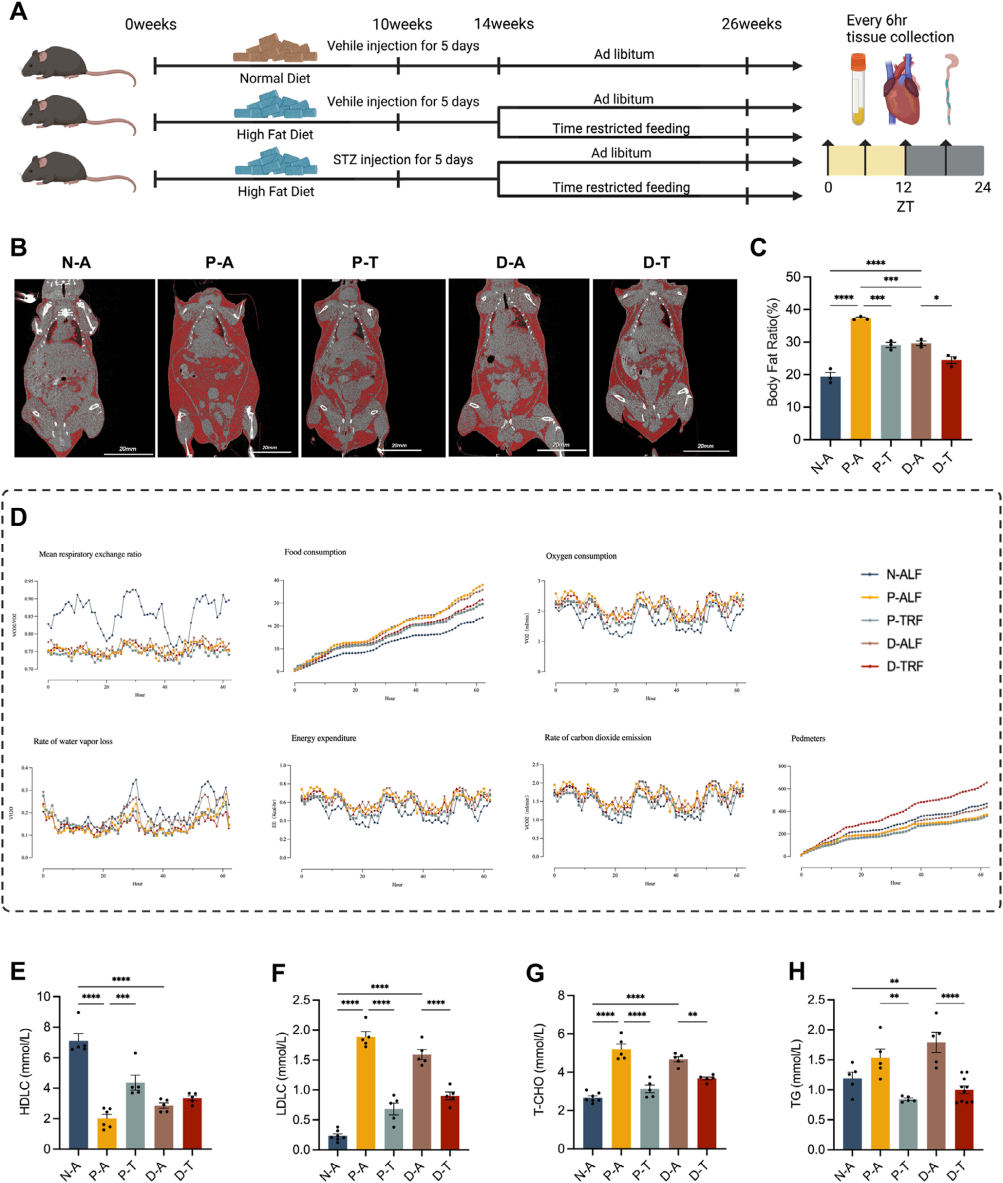
TRF ameliorates metabolic dysfunction and reduces adiposity in prediabetic mice. (A) Schematic diagram illustrating the experimental design, including animal group assignments, chronological timeline of interventions, and the 24‐h sampling schedule. (B and C) Representative images of positron emission tomography (PET) images (B) and quantitative analysis of body fat percentage (C) in mice from all groups (*n* = 3). (D) Metabolic cage recordings, including respiratory exchange ratio, food intake, oxygen consumption, water vapor loss, energy expenditure, carbon dioxide production, and locomotor activity (*n* = 3). (E–H) Quantification of serum lipid profiles, including high‐density lipoprotein cholesterol (HDL‐C, E), low‐density lipoprotein cholesterol (LDL‐C, F), total cholesterol (T‐CHO, G), and triglycerides (TG, H) (*n* = 5–10). Data are presented as mean ± SEM. *p < 0.05, **p < 0.01, ***p < 0.001, ****p < 0.0001. ZT, zeitgeber time; ZT0 indicates the onset of the light phase (08:00), and ZT12 indicates the onset of the dark phase (20:00). Consistent color‐coding is used to distinguish the experimental groups: dark blue for N‐ALF, orange for P‐ALF, light green for P‐TRF, brown for D‐ALF, and dark red for D‐TRF.

To investigate the effects of TRF on systemic metabolism across different disease stages, we established prediabetic and diabetic mouse models induced by a high‐fat diet (HFD) combined with streptozotocin (STZ). The experimental timeline and grouping schematic are presented in Figures 1A and S1A, respectively (detailed protocols are provided in *Materials and Methods* section).

As shown in Figures 1A and S1A, mice were assigned to five groups: (1) N‐ALF/N‐A (normal diet, ad libitum); (2) P‐ALF/P‐A (prediabetic, ad libitum); (3) P‐TRF/P‐T (prediabetic, TRF); (4) D‐ALF/D‐A (diabetic, ad libitum); and (5) D‐TRF/D‐T (diabetic, TRF). Body weight, blood glucose, and food intake were monitored weekly, and systemic metabolic evaluations were performed at the end of the study.

The cumulative food intake was nearly identical across all groups (Figure S1H,I). Although the TRF groups were restricted to a specific feeding window during the dark phase, their total 24‐h caloric consumption remained consistent with their respective ad libitum feeding (ALF) counterparts (Figure S1H). Despite similar food intake, the physical size of P‐ALF mice was markedly larger than that of the N‐ALF and P‐TRF groups (Figure S1B). Positron emission tomography (PET) scans further confirmed that adiposity was significantly higher in all experimental groups compared with the N‐ALF group; however, TRF intervention notably reduced body fat in both prediabetic and diabetic mice (Figure 1B,C). Serum biochemical analysis revealed that TRF significantly improved most lipid profiles, including total cholesterol (T‐CHO), triglycerides (TG), and low‐density lipoprotein cholesterol (LDL‐C). Notably, high‐density lipoprotein cholesterol (HDL‐C) levels showed no significant reduction in the diabetic group (Figure 1E–H).

To further evaluate metabolic dynamics, we monitored mice (*n* = 3 per group) using metabolic cages (Figures 1D and S1G), which revealed significant differences in 24‐h metabolic oscillations. Specifically, the N‐ALF group exhibited clear and regular diurnal fluctuations, with oxygen consumption (VO_2_), energy expenditure (EE), and physical activity peaking during the dark phase and declining during the light phase; the respiratory exchange ratio (RER) cycled periodically between 0.7 and 1.0. In the P‐ALF group, while basal metabolic rhythms were preserved, the amplitude of oscillations was attenuated, particularly with a reduced decline in RER during the light phase. In contrast, the P‐TRF group showed a marked enhancement in the rhythmicity of all metabolic parameters without altering total caloric intake. Their EE and physical activity were highly concentrated within the dark‐phase feeding window and rapidly dropped to baseline during the light‐phase fasting period. The D‐ALF group exhibited severe disruption or total loss of diurnal metabolic rhythms, characterized by flattened VO_2_ and EE curves and a persistently low RER lacking distinct phase fluctuations. Although the D‐TRF group was subjected to the same feeding constraints, their metabolic regulatory response during the nonfeeding window remained weak, failing to recover the rhythmic amplitude observed in the P‐TRF group.

Collectively, these data indicate that despite similar caloric intake, mice at different disease stages exhibit distinct metabolic profiles and oscillatory patterns. This suggests that the benefits of TRF do not stem from caloric restriction but rather from the optimization of energy distribution via the remodeling of feeding phases. While TRF effectively restores metabolic rhythmicity in the prediabetic stage, this remodeling efficacy is significantly diminished as the disease progresses to overt diabetes, likely due to profound metabolic rigidity.

### 2.2 TRF Alleviates Cardiac Senescence and Mitochondrial Damage in Prediabetic Mice but Is Ineffective at the Diabetic Stage

To investigate the cardioprotective effects of TRF against senescence, we conducted a systematic evaluation of cardiac function, histopathology, and ultrastructural integrity at the experimental endpoint.

**FIGURE 2:**
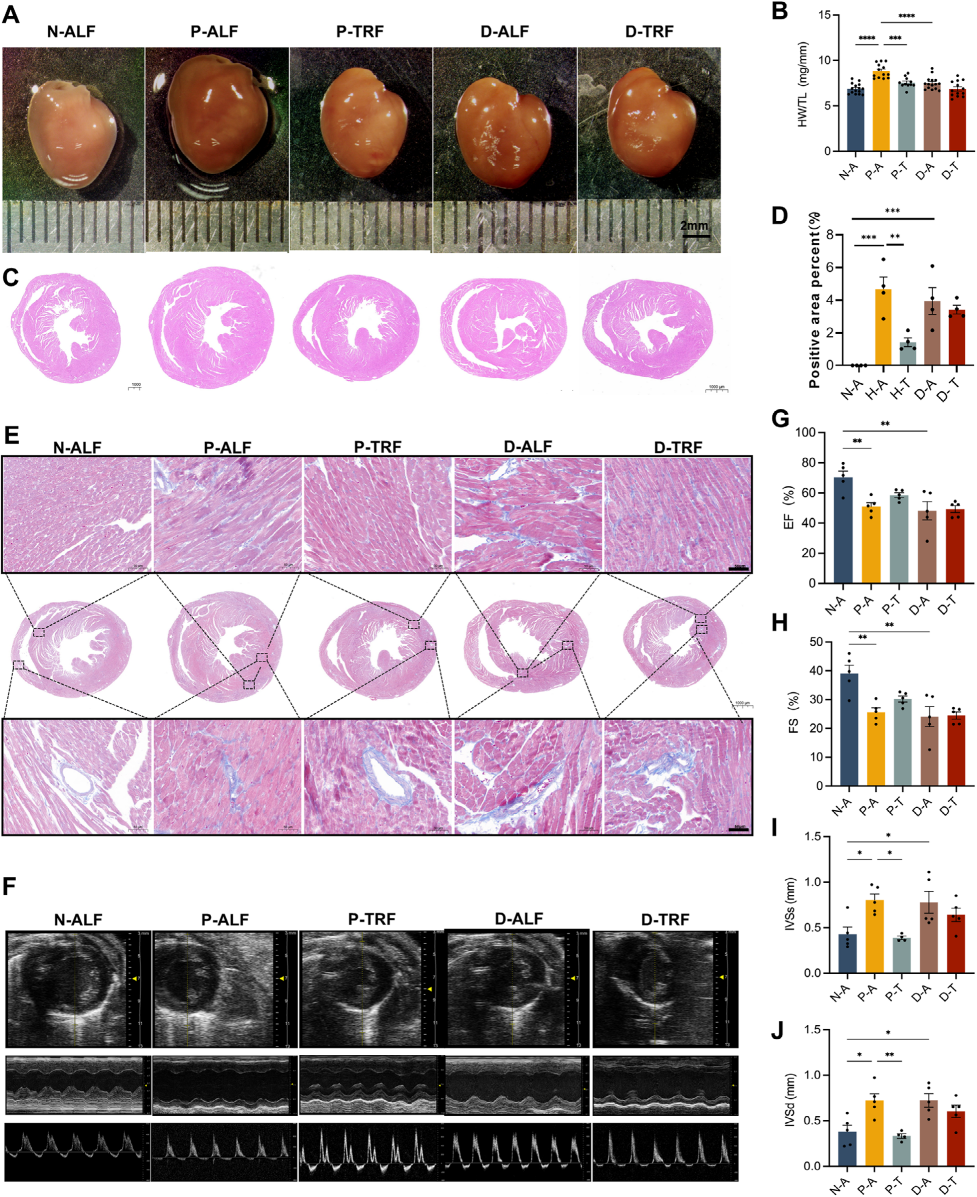
TRF alleviates cardiac senescence and mitochondrial damage in prediabetic mice but is ineffective at the diabetic stage. (A) Representative gross morphology of hearts (scale bar = 2 mm). (B) Quantitative analysis of heart weight to tibial length (HW/TL) ratio (*n* = 11–15). (C) Representative images of H&E‐stained myocardial short‐axis cross‐sections (scale bar = 1000 µm). (D and E) Representative images and quantification of Masson's trichrome staining. The middle panel shows whole myocardial cross‐sections (scale bar = 1000 µm). High‐magnification images of interstitial fibrosis (top panel) and perivascular fibrosis (bottom panel) are displayed (scale bar = 50 µm). (F) Representative echocardiographic images, including left ventricular (LV) long‐axis B‐mode images, M‐mode dimensions, and Doppler flow imaging of diastolic mitral inflow and mitral annular velocities. (G–J) Quantitative echocardiographic parameters: LV ejection fraction (EF, G), LV fractional shortening (FS, H), interventricular septum thickness in systole (IVSs, I), and diastole (IVSd, J) (*n* = 6–8). Data are presented as mean ± SEM. *p* < 0.05, **p < 0.01, ***p < 0.001, ****p < 0.0001. Consistent color‐coding is used to distinguish the experimental groups: dark blue for N‐ALF, orange for P‐ALF, light green for P‐TRF, brown for D‐ALF, and dark red for D‐TRF.

Echocardiographic assessment (Figure 2F–J) revealed that in the prediabetic stage, TRF significantly reduced the interventricular septum thickness in both systole and diastole (IVSs and IVSd) compared with the P‐ALF group. Although the left ventricular (LV) ejection fraction (EF) and fractional shortening (FS) did not reach statistical significance, a favorable improvement trend was observed in the P‐TRF group. Furthermore, the heart weight‐to‐tibial length (HW/TL) ratio was markedly elevated in the P‐ALF group relative to the control; TRF intervention successfully reversed this hypertrophic phenotype in prediabetic mice, whereas no such restoration was observed in the diabetic (D‐TRF) group (Figure 2A,B). Additional echocardiographic parameters (Figure S2A) indicated that TRF also facilitated varying degrees of improvement in the LV internal diameter in systole and diastole (LVIDd), Tei index, LV anterior wall thickness (LVAWd, LVAWs), and posterior wall thickness (LVPWd, LVPWs) specifically in prediabetic mice.

**FIGURE 3:**
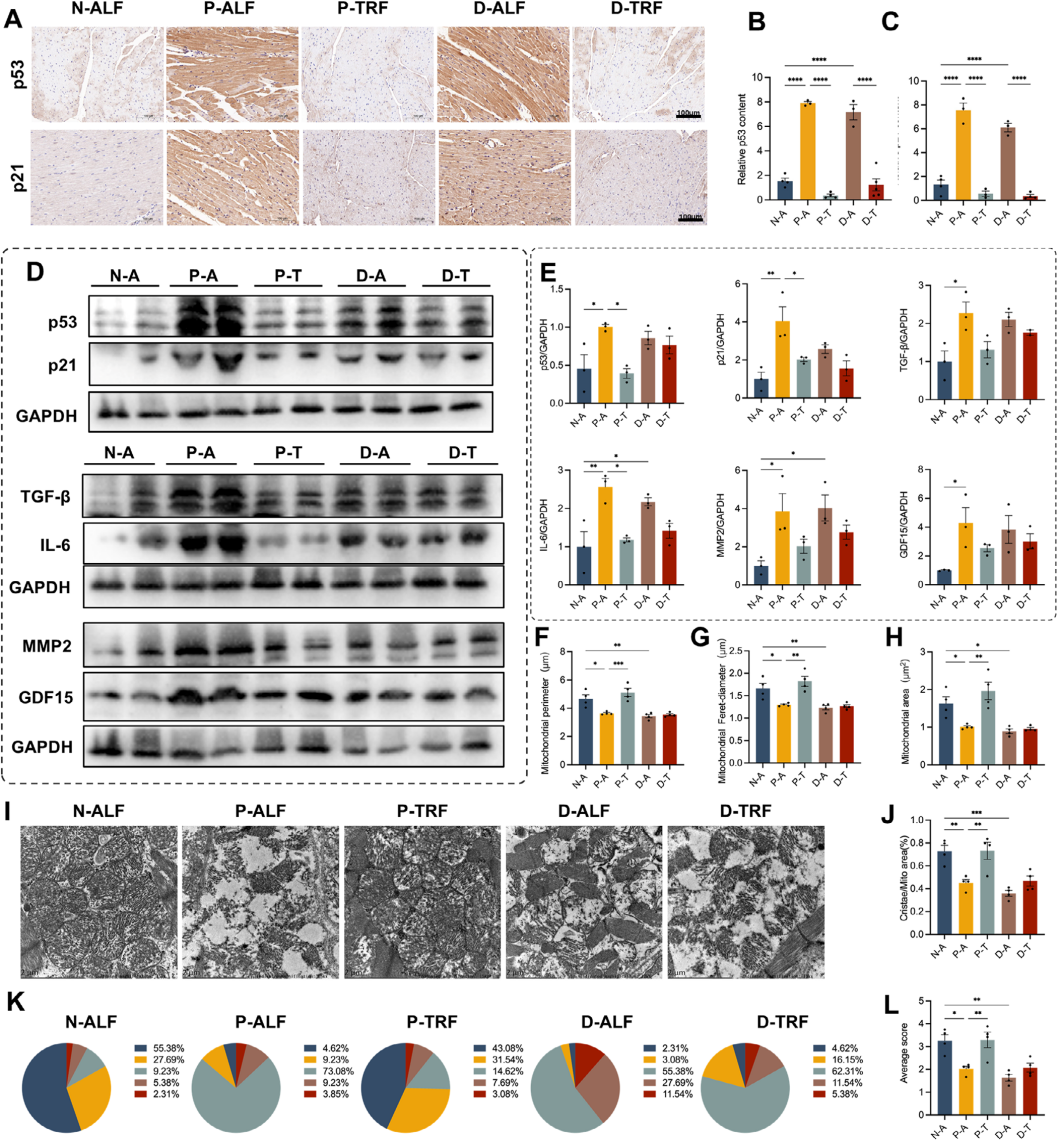
TRF reduces cardiac senescence markers and preserves mitochondrial ultrastructure in prediabetic mice but shows limited efficacy at the diabetic stage. (A–C) Representative immunohistochemistry (IHC) images and quantification of p53 and p21 (*n* = 3–5) in heart sections; scale bar = 100 µm. (D and E) Representative immunoblotting (IB) analysis and quantification of senescence markers p53/p21 and SASPs (IL‐6, TGF‐β, MMP2, GDF15) in heart tissues (*n* = 3). GAPDH was used as the loading control. (I) Representative transmission electron microscopy (TEM) images of myocardial mitochondrial ultrastructure. Scale bar = 0.5 µm. Six to ten random fields were analyzed per heart sample. (F–H, J) Quantitative morphometric analysis of mitochondria, including perimeter (F), Feret diameter (G), average mitochondrial area (H), and the ratio of cristae area to total mitochondrial area (J) (*n* = 5). (K and L) Qualitative and quantitative assessment of mitochondrial morphology (K). Frequency distribution of individual mitochondrial scores across experimental groups (L) (*n* = 4). Data are presented as mean ± SEM. *p < 0.05, **p < 0.01, ***p < 0.001, ****p < 0.0001. Consistent color‐coding is used to distinguish the experimental groups: dark blue for N‐ALF, orange for P‐ALF, light green for P‐TRF, brown for D‐ALF, and dark red for D‐TRF.

Histological evaluation via Masson's trichrome staining demonstrated prominent blue collagen deposition in the myocardial interstitium and perivascular regions of both P‐ALF and D‐ALF groups. Following TRF treatment, the fibrotic area in the P‐TRF group was significantly reduced (Figure 2D,E). Moreover, immunohistochemistry (IHC) (Figure 3A–C) and Western blot analysis (Figure 3D,E) revealed that the expression of senescence markers p53 and p21 was elevated in both P‐ALF and D‐ALF hearts compared with the N‐ALF group. TRF treatment led to a significant downregulation of these proteins. A similar trend was observed for senescence‐associated secretory phenotypes (SASPs), including IL‐6, IL‐8, TGF‐β, MMP2, and GDF15.

Furthermore, transmission electron microscopy (TEM) was employed to observe the ultrastructure of myocardial organelles (Figure 3F–L). We found that sarcomere alignment was disrupted and mitochondrial cristae were disorganized in prediabetic or diabetic mice. However, in mice receiving TRF, parameters such as mitochondrial perimeter, diameter, mean cross‐sectional area, and the cristae‐to‐mitochondrial area ratio were all improved. Using a modified five‐grade scoring system [23, 24] to quantify diabetes‐induced mitochondrial morphological damage (characterized by swelling, irregular membrane curvature, and cristae loss; Figure S2E), we observed that TRF significantly mitigated mitochondrial injury in P‐TRF mice, whereas this protective effect was not evident in D‐TRF mice.

These experimental observations demonstrate that TRF confers substantial protection to the prediabetic heart at the morphological, histological, and ultrastructural levels. However, its therapeutic efficacy is markedly limited once the disease progresses to overt diabetes, suggesting that the cardioprotective benefits of TRF are highly dependent on the metabolic stage of the disease.

### 2.3 The Benefits of TRF Are Closely Linked to Diurnal Rhythms and Autophagy Pathways

To further explore the underlying pathways mediating the cardioprotective effects of TRF, we performed RNA sequencing (RNA‐seq) on heart tissues from four randomly selected mice per group.

**FIGURE 4:**
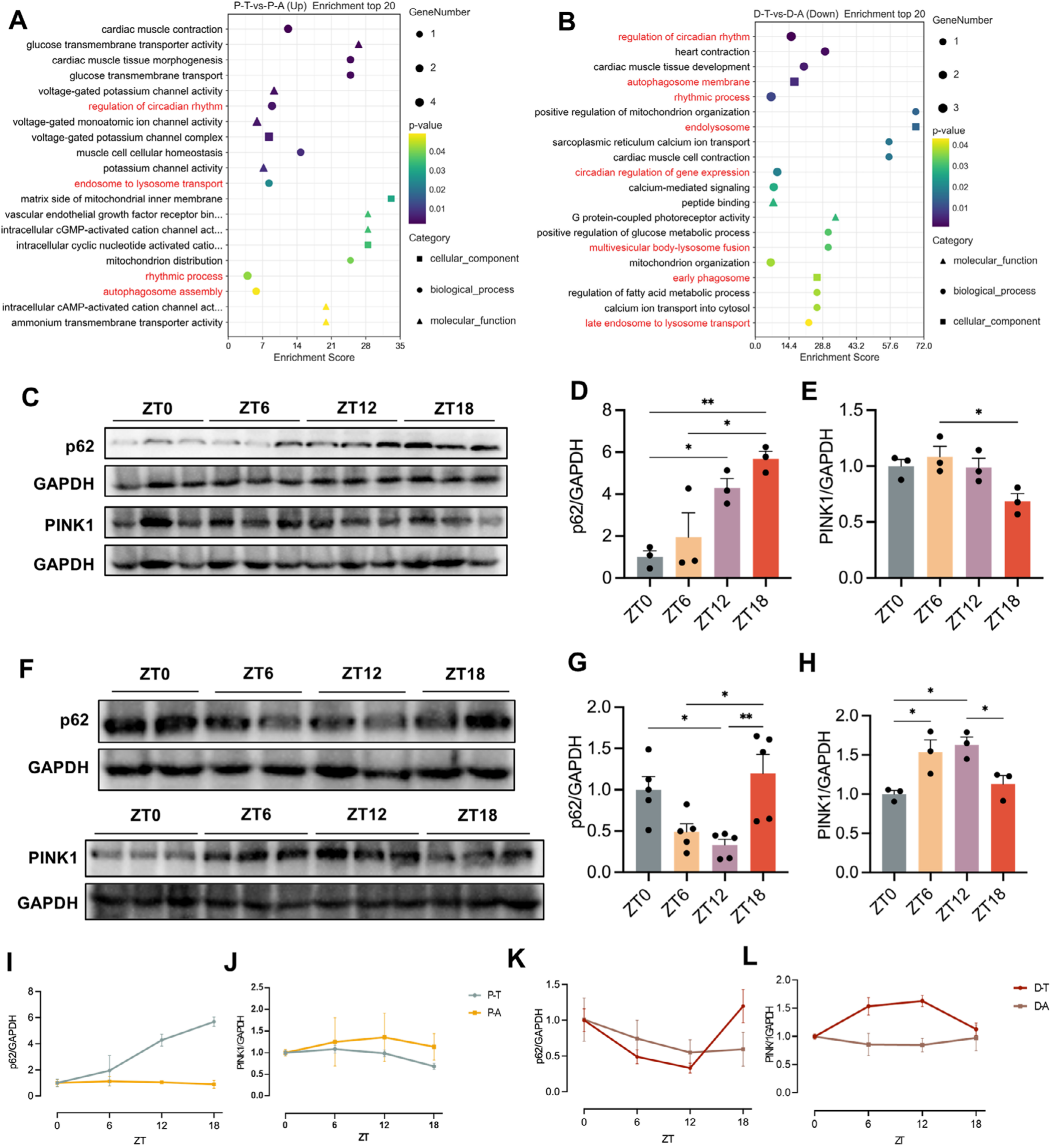
The benefits of TRF are closely linked to diurnal rhythms and autophagy pathways. (A and B) Gene Ontology (GO) biological process enrichment analysis of differentially expressed genes (DEGs) in the hearts of prediabetic (A) and diabetic (B) mice (TRF vs. ALF). Significant enrichment is observed in pathways related to rhythmic processes, circadian regulation, and autophagosome assembly. (C–E, I, and J) Representative Western blots and statistical quantification of p62 and PINK1 protein levels in the hearts of P‐TRF mice across four zeitgeber time (ZT) points. GAPDH was used as the internal loading control (*n* = 3–5). (F–H, K, and L) Representative Western blots and statistical quantification of p62 and PINK1 protein levels in the hearts of D‐TRF mice across four zeitgeber time (ZT) points. GAPDH was used as the internal loading control (*n* = 3–5). Data are presented as mean ± SEM. *p < 0.05, **p < 0.01, ***p < 0.001, ****p < 0.0001. ZT, zeitgeber time; ZT0 represents the onset of the light phase (08:00), and ZT12 represents the onset of the dark phase (20:00).

Volcano plots illustrated that TRF significantly upregulated 153 genes and downregulated 187 genes in prediabetic mice (Figure S3A). Gene Ontology (GO) enrichment analysis revealed that the top upregulated biological processes in P‐TRF mice included transporter activity, regulation of diurnal rhythm, endosome‐to‐lysosome transport, rhythmic processes, and autophagosome assembly (Figures 4A and S3B). In the diabetic groups, 195 genes were upregulated and 138 genes were downregulated when comparing D‐TRF and D‐ALF mice (Figure S3C). Notably, GO enrichment indicated that D‐TRF mice exhibited significant downregulation of biological processes such as regulation of diurnal rhythm, rhythmic processes, diurnal rhythm‐dependent regulation of gene expression, multivesicular body–lysosome fusion, late endosome‐to‐lysosome transport, autophagosome membrane, and endolysosomes (Figure 4B). Kyoto Encyclopedia of Genes and Genomes (KEGG) pathway enrichment further showed that insulin resistance and longevity‐regulating pathways were significantly attenuated in diabetic mice following TRF intervention (Figure S3E,F). These unbiased clustering approaches reveal that biological processes related to diurnal rhythm regulation and downstream autophagy are closely associated with the benefits conferred by TRF.

To investigate how TRF influences cardiac autophagic rhythms, we analyzed the dynamic changes of the key autophagy marker p62 and the mitophagy‐related protein PTEN‐induced kinase 1 (PINK1) over a 24‐h period across four zeitgeber time (ZT) points using Western blot and immunofluorescence (IF). In the prediabetic (P‐TRF) group, heart tissues exhibited robust and regular diurnal oscillations: p62 expression peaked at ZT18 and reached its nadir at ZT0, indicating efficient substrate degradation, while PINK1 showed an alternating peak near ZT6 (Figure 4C–E,I,J). In situ IF staining of cardiomyocytes (cardiac troponin T [cTnT]‐positive areas) corroborated these findings (Figure S4A,C,D), showing that the p62 signal significantly diminished at ZT0, reflecting intense activation of autophagic flux during specific intervals. In contrast, the diabetic (D‐TRF) group displayed a markedly different profile (Figures 4F–H,K,L and S4B,E,F). Although oscillatory trends remained detectable, the overall baseline expression of p62 was significantly elevated, and the depth of degradation at ZT0 was far less thorough than that in the P‐TRF group. Furthermore, the oscillatory phase of PINK1 was shifted (peaking near ZT12), and the fluorescence distribution appeared more fragmented with a significantly reduced amplitude. Data for the remaining groups (P‐ALF, D‐ALF, and N‐ALF) are provided in the supplementary materials (Figure S5).

Collectively, these findings demonstrate that the remodeling of cardiac autophagic rhythms by TRF is highly dependent on the disease stage. In the prediabetic stage, TRF establishes a robust diurnal rhythm for autophagy and mitophagy, providing significant cardioprotection by facilitating the clearance of damaged proteins and dysfunctional mitochondria at specific time points (e.g., near ZT0). However, once the disease progresses to overt diabetes, the systemic “metabolic rigidity” prevents the heart from restoring normal autophagic oscillatory phases and clearance efficiency, even under TRF intervention. This provides a molecular explanation for why TRF is highly effective against cardiac senescence in the prediabetic stage but offers limited benefits for the advanced diabetic heart.

### 2.4 TRF Restores Peripheral SCFA Rhythmicity Following Disease‐Associated Impairment of Propionate and Butyrate Metabolism

**FIGURE 5:**
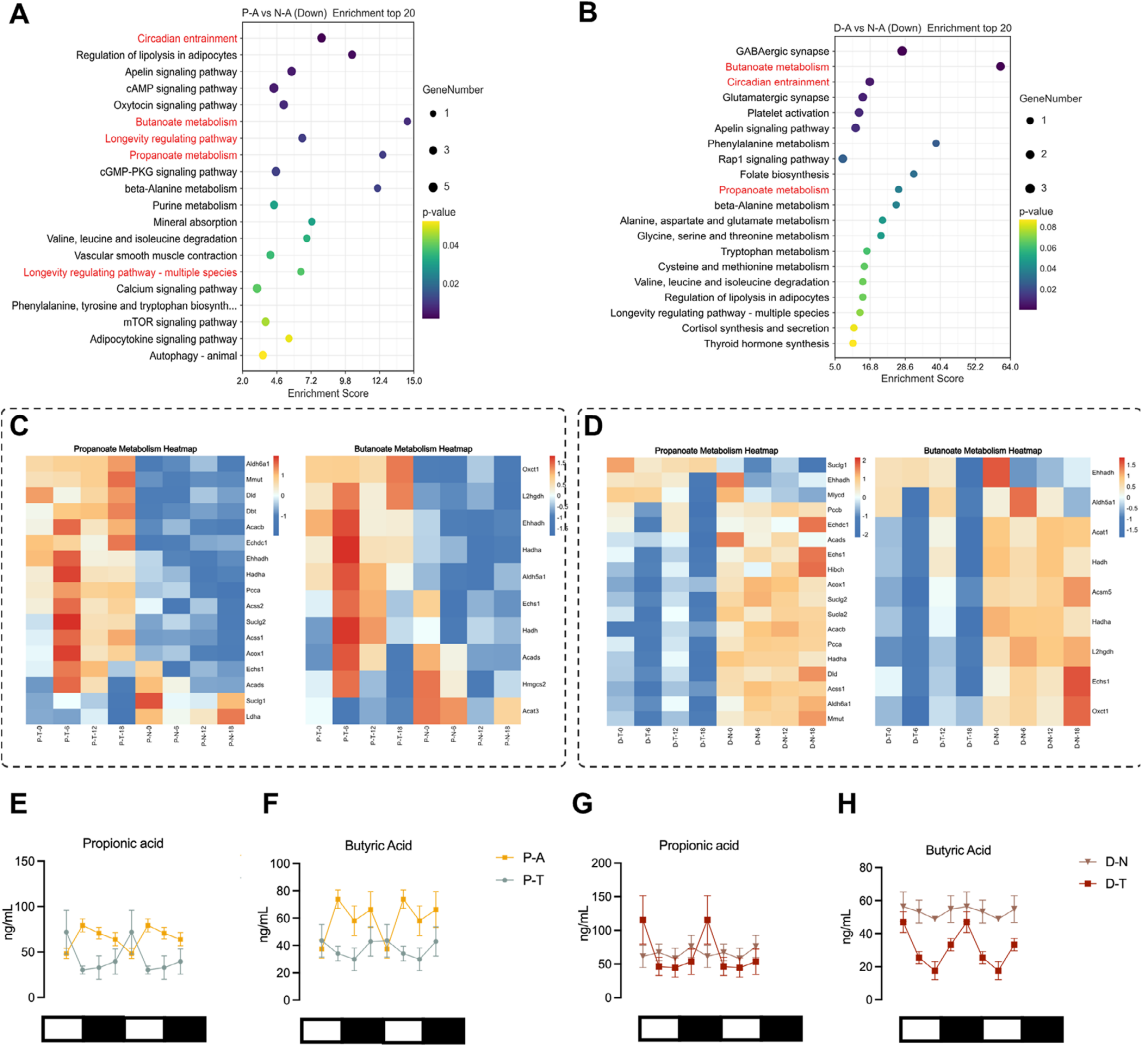
TRF restores peripheral SCFA rhythmicity following disease‐associated impairment of propionate and butyrate metabolism. (A) KEGG pathway enrichment analysis of downregulated pathways in prediabetic ALF mice compared with normal diet ALF mice (P‐ALF vs. N‐ALF). Panel (A) was used to identify metabolic pathways impaired under prediabetic conditions, thereby providing the rationale for focusing on propionate and butyrate metabolism in subsequent analyses. (B) Heatmap showing the differential expression of top‐ranked genes involved in propanoate and butanoate metabolism in the hearts of prediabetic TRF versus ALF mice. (C) KEGG pathway enrichment analysis of downregulated pathways in diabetic ALF mice compared with normal diet ALF mice (D‐ALF vs. N‐ALF). Panel (C) was used to identify metabolic pathways impaired under diabetic conditions, thereby further supporting the focus on propionate and butyrate metabolism. (D) Heatmap showing the differential expression of top‐ranked genes involved in propanoate and butanoate metabolism in the hearts of diabetic TRF versus ALF mice. (E–H) Circadian profiles of serum SCFA concentrations quantified by LC–MS/MS across four ZT points. Prediabetic mice: Temporal oscillations of serum acetate, propionate, and butyrate are shown in panels (E) and (F) (*n* = 3). Diabetic mice: Temporal oscillations of the corresponding SCFAs are shown in panels (G) and (H) (*n* = 4–7). The white and black bars at the bottom of the graphs represent the light and dark phases, respectively. Data characterize the rhythmic fluctuations of microbial metabolites and their synchronization with the TRF intervention. Data are presented as mean ± SEM. *p < 0.05, **p < 0.01, ***p < 0.001, ****p < 0.0001. ZT, zeitgeber time; ZT0 indicates the onset of the light phase (08:00), and ZT12 indicates the onset of the dark phase (20:00).

To investigate the upstream drivers of TRF‐mediated cardiac autophagic rhythm remodeling, we analyzed the differentially expressed genes (DEGs) between P‐ALF and N‐ALF groups. This analysis revealed that, in addition to the disruption of diurnal rhythms and longevity regulation signaling, the propionate and butyrate metabolism pathways were significantly downregulated. In contrast, TRF intervention in prediabetic mice significantly upregulated genes associated with SCFA metabolism in the P‐TRF group. These genes exhibited a distinct diurnal rhythm trend, with peak expression occurring at ZT6 (Figure 5A,C,E,F). Conversely, in the D‐TRF group, propionate and butyrate metabolism was downregulated, most notably at ZT6 and ZT12, representing a trend diametrically opposed to that observed in P‐TRF mice (Figure 5B,D,G,H).

The rhythmic upregulation of SCFA utilization pathways in the heart transcriptome suggested that this local metabolic remodeling might stem from fluctuations in corresponding circulating metabolites. Given that the gut microbiota is the primary source of peripheral SCFAs, we analyzed fecal samples using 16S ribosomal RNA (16S rRNA) sequencing to characterize the structure and rhythmicity of the intestinal microecology. The 16S rRNA sequencing revealed that TRF significantly influenced the gut microbial community structure. In the prediabetic stage, the Shannon diversity index showed a declining trend in the P‐TRF group compared with P‐ALF, whereas minimal differences were observed between D‐TRF and D‐ALF groups (Figure S6). At the genus level, TRF markedly induced an increase in the abundance of SCFA‐producing genera, such as Alloprevotella, Roseburia, and Blautia, and conferred them with clear diurnal oscillations, peaking at ZT18, ZT12, and ZT6, respectively (Figure S7A,B). In sharp contrast, the abundance of these SCFA‐producing genera remained consistent between D‐ALF and D‐TRF mice, showing no significant rhythmic recovery (Figure S7C,D). Additionally, several beneficial genera, including Lactobacillus and Bifidobacterium, demonstrated remodeled distribution patterns under TRF intervention (Figure S7A,B).

To validate the connection between gut microbes and cardiac function, we quantified serum SCFA levels using liquid chromatography–tandem mass spectrometry (LC–MS/MS). The results showed that serum propionate in the P‐TRF group exhibited prominent rhythmic fluctuations, which were inversely correlated with the oscillations of the autophagic substrate p62 in the heart (Figure 5E,F). In diabetic mice, however, the D‐TRF group failed to establish a rhythmic pattern tightly coupled with cardiac autophagy, despite minor fluctuations (Figure 5G,H).

These experimental findings reveal a potential upstream mechanism for TRF‐mediated cardioprotection: TRF successfully restores the diurnal oscillations of the gut microbiota and specifically enriches SCFA‐producing species in the prediabetic stage. These rhythmically fluctuating SCFAs enter the peripheral circulation as key signaling molecules, upregulating cardiac SCFA utilization and inducing rhythmic autophagy. The rhythmic remodeling of this “gut microbiota–plasma metabolite–cardiac autophagy” axis appears to be a core biological foundation by which TRF delays cardiac senescence in the prediabetic stage.

### 2.5 SCFAs Alleviate High Glucose‐Induced Cardiomyocyte Senescence by Activating Autophagic Flux

To validate the gut–heart axis effects observed in vivo, we established an in vitro myocardial senescence model using high glucose (HG) and performed interventions with SCFAs, primarily propionate and butyrate.

**FIGURE 6:**
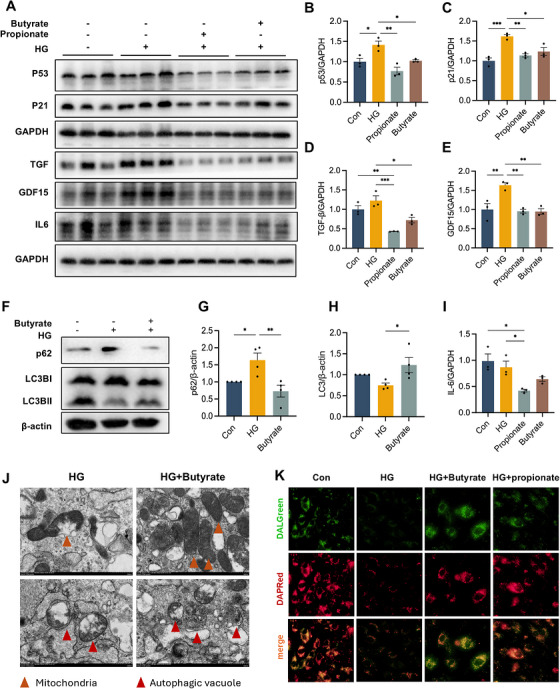
SCFAs alleviate high glucose‐induced cardiomyocyte senescence by activating autophagic flux. (A–E, I) Immunoblotting (IB) analysis and quantification of senescence markers p53, p21, and SASPs in neonatal rat cardiomyocytes (NRCMs) subjected to high glucose (HG) and treated with propionate or butyrate (1 mM) (*n* = 3). GAPDH served as loading controls for the butyrate and propionate experimental groups, respectively. (F–H) Representative IB images and quantitative analysis of autophagy markers p62 and LC3‐II/I in HG‐induced senescent NRCMs following butyrate treatment (*n* = 3). β‐Actin served as loading controls for the butyrate experimental groups, respectively. (J) Representative transmission electron microscopy (TEM) images showing mitochondria and autophagy‐related structures in HG‐induced senescent NRCMs treated with or without butyrate. Arrowheads indicate mitochondria (Mito) and autophagic vacuoles (AV). (K) Detection of autophagic flux in HG‐induced senescent NRCMs. Representative immunofluorescence images showing autophagosomes (DAPRed, red) and autolysosomes (DALGreen, green) in NRCMs exposed to high glucose (HG) and treated with or without propionate and butyrate (1 mM). Data are presented as mean ± SEM. *p < 0.05, **p < 0.01, ***p < 0.001, ****p < 0.0001.

Western blot analysis showed that the levels of senescence markers p53 and p21, as well as SASPs such as TGF‐β, GDF15, and IL‐6, were significantly elevated in the HG group. However, treatment with either propionate or butyrate markedly downregulated the expression of p53, p21, and SASPs, with propionate exhibiting a more potent antisenescence effect than butyrate (Figure 6A–E,G). Furthermore, senescence‐associated β‐galactosidase (SA‐β‐gal) staining in primary cardiomyocytes revealed a significant increase in the percentage of positive cells in the HG group, which was effectively reduced by butyrate intervention (Figure S8A).

After confirming that in vitro SCFA intervention improves markers of myocardial senescence and based on our RNA‐seq findings suggesting that SCFAs likely act through autophagy (refer to the earlier result: The benefits of TRF are closely linked to diurnal rhythms and autophagy pathways), we further examined the autophagic pathways at the cellular level. Western blot analysis of HG‐treated cardiomyocytes following propionate or butyrate intervention revealed that p62 expression levels were markedly increased in the HG group, while LC3BII levels were concurrently elevated, indicating impaired autophagic flux. Upon the addition of propionate or butyrate, p62 levels declined and LC3B‐II levels decreased, suggesting that SCFAs can activate autophagy (Figures 6F–H and S8B–D). To further determine whether SCFAs exert their effects by enhancing autophagic flux, we utilized Bafilomycin A1 (Baf A1), a vacuolar H^+^‐ATPase inhibitor that blocks lysosomal degradation. In the presence of Baf A1, treatment with propionate or butyrate still significantly altered the accumulation levels of p62 and LC3B‐II, supporting the ability of SCFAs to enhance autophagic flux (Figure S8E–J).

In parallel, we performed TEM and specialized fluorescence imaging to evaluate the impact of SCFAs on mitochondrial morphology and autophagic flux. TEM results showed that HG treatment led to a substantial increase in mitochondrial fragmentation, whereas butyrate treatment resulted in more intact mitochondrial structures and typical autophagosome sequestration (Figure 6J). To further visualize the autophagic process, we utilized DALGreen and DAPRed probes to detect autolysosomes and autophagosomes, respectively. As shown in Figure 6K, HG exposure significantly suppressed the formation of acidic autolysosomes. However, treatment with propionate or butyrate markedly increased both the fluorescence intensity and the number of puncta for DALGreen and DAPRed, demonstrating that SCFAs enhance autophagosome formation and subsequent fusion with lysosomes. These findings visually confirm that SCFAs effectively restore and promote autophagic flux, thereby antagonizing HG‐induced cellular senescence.

These in vitro experimental results support the role of SCFAs as key metabolic mediators of TRF‐induced effects. By directly activating autophagic flux, promoting autophagosome–lysosome fusion, and maintaining mitochondrial integrity, SCFAs directly antagonize HG‐induced cellular senescence, providing mechanistic support for the systemic observations.

### 2.6 p62 Is a Pivotal Molecular Switch Mediating the Cardioprotective Effects of TRF

**FIGURE 7:**
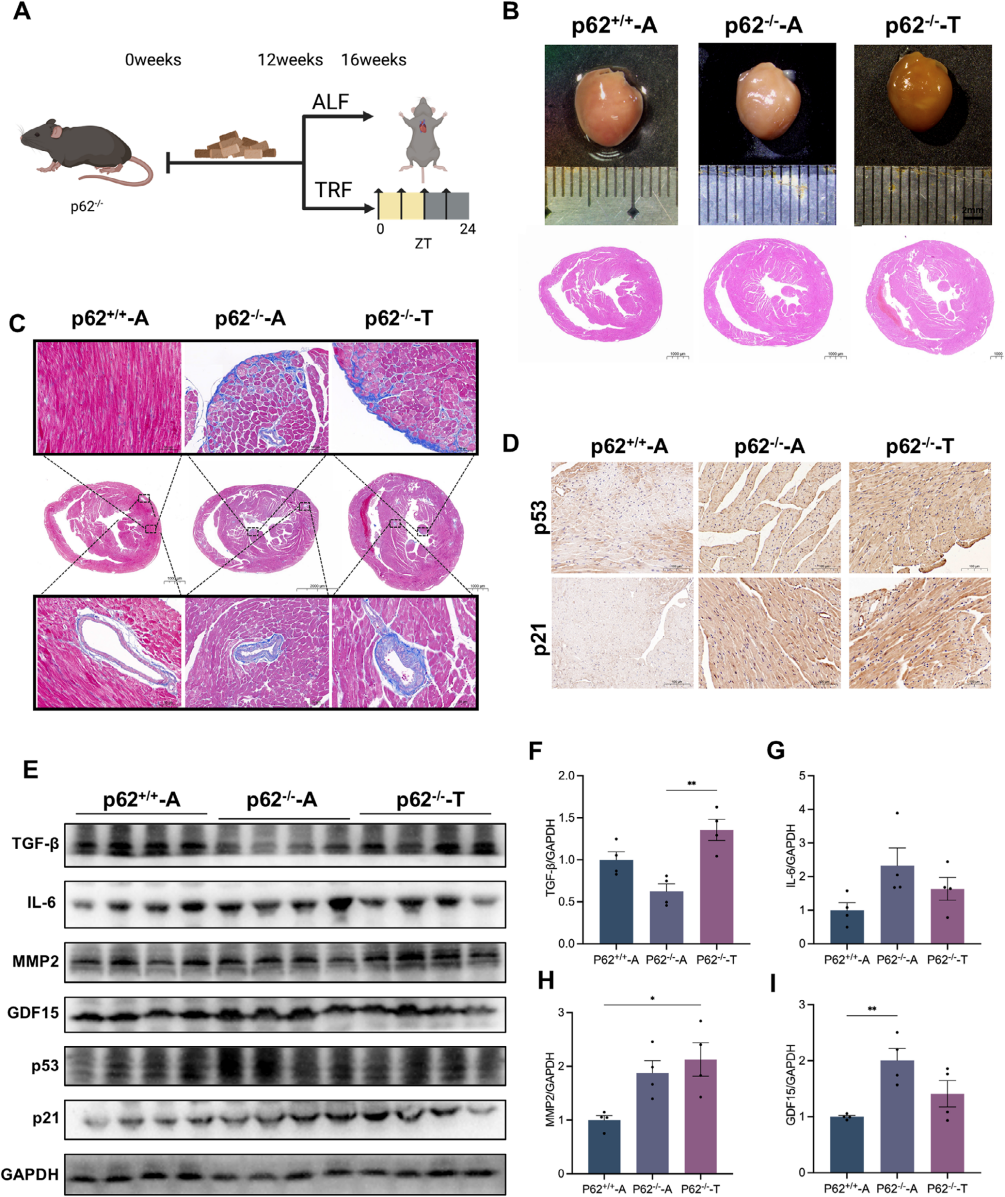
p62 deficiency attenuates the TRF‐mediated improvement in cardiac remodeling and senescence. (A) Schematic diagram illustrating the experimental design, including animal grouping, intervention timelines, and the tissue collection schedule. (B) Representative gross morphology of hearts (scale bar = 2 mm, B) and H&E‐stained myocardial cross‐sections (scale bar = 1000 µm, C). (C) Representative images and quantitative analysis of Masson's trichrome staining. The middle panel shows whole myocardial cross‐sections (scale bar = 1000 µm). High‐magnification images of interstitial fibrosis (top panel) and perivascular fibrosis (bottom panel) are provided (scale bar = 50 µm). (D) Representative immunohistochemistry (IHC) images showing p53 and p21 expression levels in heart tissues (scale bar = 100 µm). (E–I) Representative immunoblotting images and proteins quantitative analysis of SASPs (TGF‐β, IL‐6, MMP‐2, GDF15) in the myocardium (*n* = 4). GAPDH was used as the loading control. Data are presented as mean ± SEM. *p < 0.05, **p < 0.01, ***p < 0.001, ****p < 0.0001.

**FIGURE 8:**
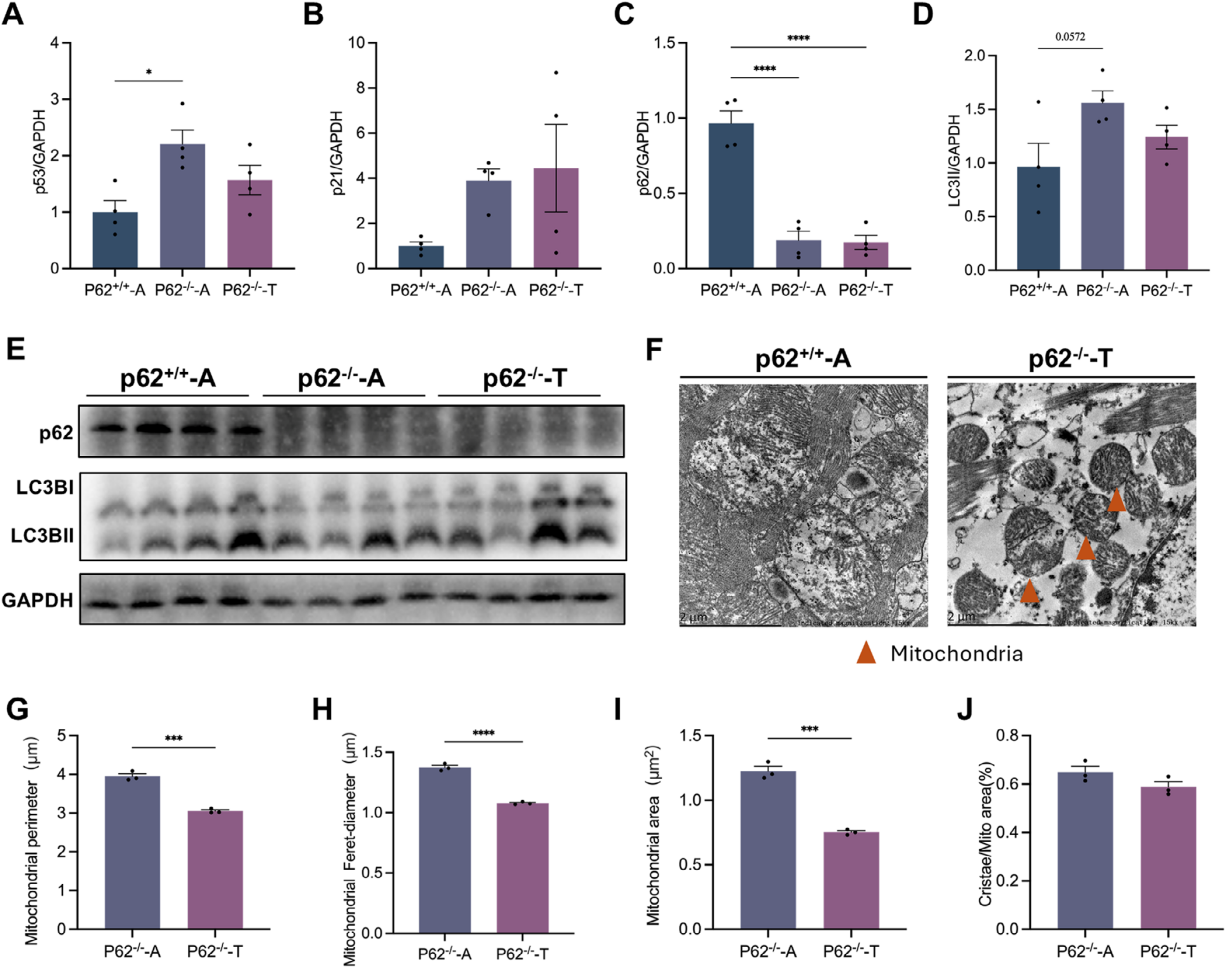
p62 deficiency compromises TRF‐induced autophagy activation and mitochondrial protection. (A and B) Quantitative analysis of p53/p21 in the myocardium (*n* = 4). GAPDH was used as the loading control. (C–E) Quantitative analysis of p62/LC3B in the myocardium (*n* = 4). GAPDH was used as the loading control. (F) presentative transmission electron microscopy (TEM) images of myocardial ultrastructure (scale bar = 0.5 µm). Arrowheads indicate mitochondria (Mito). (G–J) Morphometric quantification of mitochondrial health, including mitochondrial perimeter (G), Feret diameter (H), average mitochondrial area (I), and the ratio of cristae area to total mitochondrial area (J) (*n* = 3). Analysis was performed on 6–10 random fields per heart. Data are presented as mean ± SEM. *p < 0.05, **p < 0.01, ***p < 0.001, ****p < 0.0001.

Given our previous observation that TRF robustly drives the rhythmic oscillation of p62 in the heart, we further utilized p62‐deficient (p62^−/−^) mice to verify the necessity of this molecule in TRF‐induced benefits (Figure 7A). Our results showed that in the absence of p62, the levels of microtubule‐associated protein 1 light chain 3B‐II (LC3B‐II) were significantly higher than those in wild‐type mice, and TRF failed to reduce this LC3B‐II accumulation (Figure 8C–E). Simultaneously, the regulatory effects of TRF on cardiac morphology were abolished, with no significant differences in heart size observed among the three groups (Figure 7B). Consistent with these findings, histological Masson's trichrome staining and Western blot analysis demonstrated that TRF could not alleviate myocardial fibrosis or downregulate the expression of senescence markers such as p53 and p21 in p62^−/−^ mice (Figures 7C–I and 8A,B).

Similarly, we assessed the myocardial ultrastructure of p62^−/−^ mice using TEM. We observed that regardless of the feeding regimen (ALF or TRF), p62^−/−^ mice exhibited severe mitochondrial structural damage, characterized by mitochondrial swelling, vacuolization, and cristae disorganization. Quantitative analysis confirmed that TRF was unable to ameliorate the series of mitochondrial morphological dysfunctions induced by p62 deficiency (Figure 8F–J).

This in vivo evidence establishes that the reparative effects of TRF on cardiac senescence, fibrosis, and mitochondrial homeostasis are strictly dependent on p62. These findings confirm that p62 serves as a pivotal molecular switch that translates TRF‐induced rhythmic signals into the maintenance of cardiac homeostasis.

## 3 Discussion

TRF has emerged as a promising nonpharmacological intervention for cardiometabolic health. However, clinical and preclinical studies have reported inconsistent results regarding its antiaging efficacy. Our study provides a novel explanation for these discrepancies: the efficacy of TRF is strongly stage dependent. In prediabetic mice, TRF significantly attenuated cardiac senescence and mitochondrial dysfunction, but these benefits were largely abolished in the overt diabetes stage. This suggests that while TRF is a potent prophylactic strategy to delay premature cardiac aging during early metabolic derangement, its therapeutic capacity is limited by the “metabolic rigidity” characterized by advanced diabetes. This aligns with recent clinical observations that dietary interventions are more effective when metabolic plasticity is preserved [8, 13, 25, 26].

Mitochondrial dysfunction is a hallmark of cardiac aging, leading to the accumulation of damaged organelles and the activation of the SASP [27, 28, 29]. We demonstrated that TRF revitalizes the aged heart by restoring mitochondrial turnover. Crucially, using p62^−/−^ mice, we identified p62‐mediated autophagy as the indispensable executor of TRF's benefits. In the absence of p62, TRF failed to clear damaged mitochondria or suppress SASP markers like IL‐6 and TGF‐β. Furthermore, we observed a critical “phase‐shift” in autophagic markers: in prediabetic mice, p62 reached its nadir at ZT0, coinciding with the peak of autophagic clearance, whereas this rhythm was blunted or delayed in diabetic mice. This rhythmic “re‐synchonization” of autophagy appears to be the molecular basis for cardiac rejuvenation.

A key finding of this study is the role of the gut–heart axis in mediating TRF's effects. TRF acted as a strong entraining cue, restoring the diurnal oscillations of SCFA‐producing microbiota (e.g., *Roseburia* and *Blautia*). This microbial rhythmicity translated into cyclic fluctuations of peripheral SCFA levels, particularly propionate and butyrate. Our in vitro data confirmed that these SCFAs enhance autophagic flux and preserve mitochondrial integrity in cardiomyocytes. We propose that these rhythmically fluctuating SCFAs serve as “metabolic zeitgebers” that synchronize the cardiac peripheral clock with the feeding–fasting cycle. In diabetes, the loss of this microbial rhythmicity contributes to the failure of TRF, highlighting the gut microbiota as a potential therapeutic target to enhance the benefits of fasting‐based regimens. While our metabolomic analysis primarily highlighted the rhythmic oscillations of propionate and butyrate, it is important to note that other SCFAs, such as acetate, also play a profound role in cardiovascular regulation. As demonstrated by Poll et al. [30], acetate significantly influences blood pressure and heart rate, suggesting that a comprehensive restoration of the SCFA profile may be important for the full antiaging efficacy of TRF. Furthermore, while our use of exogenous SCFA supplementation provides strong evidence for their cardioprotective role, we acknowledge that definitive causality between TRF‐induced microbial shifts and cardiac health would be further strengthened by fecal microbiota transplantation (FMT) or germ‐free mouse studies. Future research employing FMT is necessary to confirm whether the specific rhythmicity of the microbiota is the primary driver of cardiac rejuvenation. Interestingly, the diurnal rhythmicity and overall levels of propionate and butyrate remained blunted in diabetic mice even under TRF. This suggests that the severe metabolic derangement in overt diabetes may cause an irreversible shift in the gut microbial ecosystem—a state of “microbial rigidity”—where the niche for beneficial SCFA‐producers is largely lost, rendering feeding–fasting cycles ineffective as an entraining cue.

From a clinical perspective, TRF is a highly accessible approach that does not require caloric restriction or complex nutritional expertise. Our findings suggest that the clinical condition and timing of intervention are paramount. It should be acknowledged that our diabetic model, induced by high‐dose STZ, results in substantial β‐cell loss and severe insulin deficiency. This state of “metabolic exhaustion” may represent a level of metabolic rigidity that exceeds the therapeutic threshold of TRF alone. While this severe model allowed us to test the functional limits of TRF, the lack of benefit in the D‐TRF group may specifically reflect the absence of residual insulin signaling required for nutrient‐sensing synchronization. Future studies employing partial insulin sensitization or less severe models of Type 2 diabetes are needed to determine if restoring a baseline of metabolic plasticity can “rescue” the cardioprotective efficacy of TRF in advanced disease stages. While TRF with a 10‐h window may be beneficial in early‐stage metabolic disease, the “metabolic rigidity” of advanced diabetes may require adjunctive therapies—such as SCFA supplementation or microbiota modulation—to achieve similar antiaging effects. Future research should investigate whether combining TRF with specific prebiotics can “rescue” the blunted response in the diabetic population, potentially offering a personalized approach to cardiovascular health management in the elderly.

### 3.1 Limitations of the Study

Several limitations of this study warrant consideration. First, while the HFD/STZ model is commonly used to model Type 2 diabetes, the acute β‐cell necrosis induced by STZ may not fully capture the chronic, progressive insulin resistance observed in human patients. Importantly, such profound “glucose toxicity” in the diabetic groups may bypass or overwhelm the protective effects of autophagic entrainment, potentially explaining the lack of TRF efficacy in advanced stages. Second, although we identified p62 as a key mediator, the use of global p62^−/−^ mice may introduce systemic metabolic influences; thus, future studies using cardiomyocyte‐specific models are needed to isolate heart‐intrinsic mechanisms. Third, while TRF significantly improved cardiac structure and molecular senescence markers, the lack of statistical significance in EF and FS suggests its benefits may primarily target early‐stage structural remodeling rather than acute functional recovery. Finally, the limited sample size (*n* = 3) for metabolic cage monitoring necessitates that these rhythmic metabolic shifts be interpreted as pilot observations.

## 4 Conclusions

TRF is a potent nonpharmacological strategy for delaying premature cardiac aging, specifically during the prediabetic stage. TRF acts as a powerful chronotherapeutic cue that reshapes the “gut microbiota–SCFA–cardiac p62” axis, thereby restoring the rhythmicity of autophagy and maintaining mitochondrial homeostasis. However, the efficacy of TRF is strictly stage dependent, as its benefits are largely blunted by the metabolic rigidity inherent in overt diabetes. These findings underscore the importance of early intervention and highlight the potential of targeting the gut–heart axis to protect against cardiac aging.

## 5 Materials and Methods

### 5.1 Mice

Eight‐week‐old male C57BL/6J mice (20–25 g) were obtained from Hunan SJA Laboratory Animal Co., Ltd. (Hunan, China). All animal procedures were approved by the Ethics Committee of the Second Xiangya Hospital of Central South University (Approval No. 2022400) and conducted in strict accordance with the *Guide for the Care and Use of Laboratory Animals* (NIH Publication, 8th Edition, 2011). Mice were maintained in a temperature‐controlled environment with a 12:12‐h light/dark cycle (lights on at 08:00, ZT0; lights off at 20:00, ZT12).

### 5.2 Establishment of Prediabetic and Diabetic Models

Mice were randomly assigned to either a normal diet or a HFD (60% kcal from fat; D12492; Research Diets). After 8 weeks of dietary intervention, metabolic evaluations were performed. Mice on the HFD that exhibited hyperglycemia and impaired glucose tolerance were defined as the prediabetic model [31]. To establish the diabetic model, HFD‐fed mice were intraperitoneally injected with low‐dose STZ (Sigma–Aldrich, St. Louis, MO, USA) at 40 mg/kg for 5 consecutive days. Fasting blood glucose (FBG) was monitored, and mice with FBG levels >11.0 mmol/L were considered diabetic [25, 32]. For mice that did not initially meet this threshold, additional low‐dose STZ injections were administered until the diagnostic criteria were achieved.

### 5.3 Experimental Study Design

Following model establishment, mice were assigned to five experimental groups:

1. N‐ALF/N‐A (normal diet, ad libitum);
2. P‐ALF/P‐A (prediabetic, ad libitum);
3. P‐TRF/P‐T (prediabetic, TRF);
4. D‐ALF/D‐A (diabetic, ad libitum);
5. D‐TRF/D‐T (diabetic, TRF).

As in previous studies [17, 33, 34, 35], in the TRF groups, mice were restricted to a 10‐h feeding window from 22:00 to 08:00 (ZT14 to ZT0), aligning with their natural nocturnal active phase to reinforce endogenous diurnal rhythms. During the remaining 14‐h fasting period (ZT0 to ZT14), food was removed while water remained accessible ad libitum. ALF groups had unrestricted access to food throughout the 24‐h cycle. To ensure reproducibility, two independent cohorts were used: Cohort 1 for indirect calorimetry (Week 8) and PET‐CT (Week 9); Cohort 2 for terminal assessments (Week 12). To minimize the confounding effects of diurnal variation, all nonrhythmic terminal assessments, including echocardiography, PET‐CT imaging, and baseline serum metabolic marker collection—were strictly performed during the early light phase between ZT2 and ZT4. After 12 weeks, mice were euthanized via pentobarbital anesthesia (100 mg/kg, i.p.). To capture diurnal rhythms oscillations, plasma, heart, and fecal samples were collected every 6 h (ZT0, ZT6, ZT12, and ZT18) (Figure 1A,B).

### 5.4 Echocardiography

Transthoracic echocardiography was performed using the Vevo 2100 high‐resolution imaging system (FUJIFILM VisualSonics, Toronto, Canada). Mice were anesthetized with 1.5% isoflurane and maintained in a supine position on a heated platform to stabilize the heart rate. B‐mode and M‐mode images were acquired simultaneously from the parasternal long‐axis view to evaluate LV dimensions and contractile function. Pulsed‐wave Doppler imaging of the pulmonary artery was obtained from the parasternal short‐axis view to assess right ventricular hemodynamics. All parameters were measured offline using Vevo CQ software, and data were averaged over three consecutive cardiac cycles to minimize variability.

### 5.5 Body Composition Analysis

In vivo body composition was evaluated using a high‐resolution small‐animal PET/CT system (Super Nova; PINGSENG, Shanghai, China). To quantify fat mass, lean mass, and bone mineral density, mice were anesthetized and subjected to micro‐CT scanning. The acquired images were reconstructed and analyzed using dedicated postprocessing software (PINGSENG) to determine the body composition parameters for each group.

### 5.6 Indirect Calorimetry and Metabolic Phenotyping

Whole‐body metabolic profiling was conducted using an automated multiparameter metabolic cage system (Sable Systems International, North Las Vegas, NV, USA). After a 24‐h acclimation period, mice (*n* = 3 per group) were continuously monitored for 48 h to capture high‐resolution metabolic data. The system utilized high‐precision sensors to record VO_2_, carbon dioxide emission, and the rate of water vapor loss. Based on gas exchange data, the RER and EE were calculated to evaluate metabolic flexibility and substrate utilization.

To quantify physical activity, advanced infrared motion‐tracking sensors were employed to monitor total locomotion (all‐meters), ambulatory movement (pedometers), and an index of fine activity (e.g., grooming and feeding). Additionally, food and water consumption were monitored in real‐time to assess energy and fluid intake dynamics. All metabolic and behavioral data were synchronized with the 12:12‐h light/dark cycle and normalized to body mass or effective metabolic mass where appropriate to ensure intergroup comparability.

### 5.7 Intraperitoneal Glucose Tolerance Test

As described previously [36], to evaluate systemic glucose homeostasis, an intraperitoneal glucose tolerance test was performed. Mice were fasted for 16 h (with free access to water) before receiving an intraperitoneal injection of D‐glucose (2 g/kg body weight). Blood samples were collected from the tail vein at multiple time points: 0 (baseline), 15, 30, 60, and 120 min postinjection. Blood glucose levels were measured using a handheld glucometer (Accu‐Chek; Roche Diagnostics). The area under the curve was calculated using the trapezoidal rule to assess total glucose excursion, with baseline values subtracted where indicated.

### 5.8 Fecal Microbiota 16S rRNA Analysis

Genomic DNA was extracted and amplified under the manufacturer's protocol, which served as a template of bacterial 16S rRNA genes [31]. The amplicons were then purified and quantified. Sequencing was conducted on an Illumina NovaSeq 6000 platform (Illumina Inc., San Diego, CA, USA), at OE Biotech Company (Shanghai, China).

### 5.9 Histological Analysis

Tissues from mice underwent perfusion‐fixation with 4% paraformaldehyde, paraffin embedding, and 5 µm sectioning. Sections were stained with hematoxylin and eosin (H&E) for general morphology and Masson's trichrome for collagen deposition using kits from Baiqiandu Biotechnology (China). Myocardial architecture, interstitial fibrosis, and perivascular collagen content were determined by direct pixel quantification using Image‐Pro Plus (Media Cybernetics, MD, USA). Collagen volume fraction was computed for the entire tissue section by calculating the ratio of the collagen‐positive area to the total myocardial area. Optical densities were calculated by normalizing the signal intensity within a field to the underlying area of tissue within that field.

### 5.10 Immunohistochemistry

Immunohistochemical staining was performed to assess the expression and localization of senescence markers within the myocardium. Briefly, paraffin‐embedded sections (5 µm) were deparaffinized, rehydrated, and subjected to heat‐induced antigen retrieval. After blocking endogenous peroxidase activity and nonspecific binding with 5% BSA, sections were incubated overnight at 4°C with primary antibodies against p53 (Abclonal; 1:1000) and p21 (Servicebio; 1:100). Following incubation with HRP‐conjugated secondary antibodies, the signal was visualized using a DAB substrate kit (Baiqiandu Biotechnology), and sections were counterstained with hematoxylin. Images were captured using a light microscope. The mean optical density or positive cell counts were quantified using Image‐Pro Plus (Media Cybernetics) by an investigator blinded to the experimental groups.

### 5.11 Immunofluorescence

For high‐resolution localization of autophagy and senescence markers, IF was performed. Following antigen retrieval and blocking, myocardial sections were coincubated overnight at 4°C with primary antibodies against the target proteins—PINK1 (Proteintech; 23274‐1‐AP; 1:100) or p62 (CST; D6M5X; 1:400)—and the cardiomyocyte‐specific marker cTnT (15513‐1‐AP; 1:200) for cellular identification.

Subsequently, sections were incubated with corresponding FITC or TRITC‐conjugated secondary antibodies (1:100; SeraCare, USA) for 1 h at 37°C. Nuclei were counterstained with DAPI. All fluorescence images were acquired using an Olympus confocal microscope (Japan). To ensure comparability, identical laser power, gain, and exposure times were maintained across all groups. For quantitative analysis, cTnT‐positive areas were defined as the region of interest (ROI) using ImageJ software (NIH, USA), effectively isolating signals within cardiomyocytes and excluding the cardiac interstitium or epicardial vessels. The Integrated Density and puncta counts were measured within these defined ROIs by two independent investigators blinded to the treatment allocation.

### 5.12 ELISA

Serum concentrations of inflammatory cytokines and metabolic hormones, including insulin (Thermo Fisher Scientific, USA), TGF‐β, IL‐6 (Proteintech, China), and IL‐8 (Wuhan Fine Biotech, China), were quantified using commercially available ELISA kits according to the manufacturer's protocols.

### 5.13 Serum Biochemical Assays

Lipid profiles, including T‐CHO, TG, LDL‐C, and HDL‐C, were measured using automated enzymatic assays (Nanjing Jiancheng Bioengineering Institute, Nanjing, China). All serum samples were processed in duplicate to ensure measurement accuracy.

### 5.14 Metabolomic and RNA‐Sequencing Analysis

Blood was collected from the eyeball and the serum was separated and frozen at −80°C before being sent to OE Biotech Co., Ltd. for metabolite analysis. After extraction of serum metabolites, LC–MS/MS analysis was performed. Mouse heart tissues were frozen in liquid nitrogen and stored at −80°C until RNA extraction. Transcriptome sequencing was performed by OE Biotechnology Co., Ltd.

### 5.15 Cell Culture

In vitro experiments were performed with H9C2 cells and neonatal rat cardiomyocytes (NRCMs). H9C2 cells (CL‐0089; Procell, Wuhan, China) were cultured in DMEM/F12 medium (Gibco, Waltham, MA, USA) supplemented with 10% newborn calf serum.

### 5.16 NRCM Isolation and Culture

Briefly, the hearts of neonatal rats were rapidly separated and subjected to sequential digestion using collagenase Type II (LS004176; Worthington Biochemical Corporation, Lakewood, NJ, USA) prepared in Hanks’ balanced salt solution (Gibco). Noncardiomyocytes were removed by differential preplating based on their faster adhesion, and the remaining cardiomyocyte‐enriched suspension was collected for subsequent culture. Cells were then cultured in a medium composed of 80% DMEM (Gibco), 20% newborn calf serum (Procell; 164210), supplemented with BrdU (HY‐15910; MCE), at 37°C in a humidified incubator containing 95% air and 5% CO_2_.

### 5.17 Cell Experimental Study Design

H9C2 cells or NRCMs pretreated with either 1 mM butyrate (Sigma; 303410) or propionate (Sigma; P5436) for 30 min were grown in a HG medium (37.5/25.5 mM) for 96 h. Baf A1 was added 100 nM for 2 h before cell collection. In selected experiments, cells were treated with SCFAs for 36 h. All experiments were repeated at least three times.

### 5.18 SA‐β‐Gal Staining

H9C2 cells rendered senescent by HG exposure were treated with or without intervention for the indicated durations. Following the manufacturer's instructions (C0602; Beyotime, China), cells were washed three times with PBS and fixed with the provided fixation buffer for 15 min at room temperature. Subsequently, the cells were incubated overnight at 37°C (without CO_2_) with freshly prepared SA‐β‐gal staining solution (pH 6.0). The proportion of SA‐β‐gal‐positive cells (blue‐stained) relative to the total number of cells was determined by counting at least five random fields per well.

### 5.19 Western Blotting

Samples were lysed using RIPA buffer supplemented with protease and phosphatase inhibitors (CWBIO, Jiangsu, China) on ice for at least 30 min. Protein concentrations were determined, and 20 µg of protein per sample was separated by SDS‐PAGE and electro‐transferred onto PVDF membranes (Millipore, Darmstadt, Germany).

Following blocking with 5% nonfat milk, the membranes were incubated overnight at 4°C with primary antibodies against: LC3 (Abcam; ab192890; 1:1000), p62 (CST; #39749s; 1:1000), TGF‐β (CST; #3711s; 1:1000), p53 (Proteintech; #80077‐1‐RR; 1:1000), p21 (Proteintech; #10355‐1‐AP; 1:1000), PINK1 (Proteintech; #23274‐1‐AP; 1:1000), MMP2 (Proteintech; #10373‐2‐AP; 1:1000), GDF15 (Proteintech; #27455‐1‐AP; 1:1000), GAPDH (Proteintech; #60004‐1‐Ig; 1:4000), and β‐actin (ABclonal; AC038; 1:10,000).

After washing, the membranes were incubated with HRP‐conjugated goat antirabbit or antimouse IgG (Proteintech; 1:5000) for 2 h. Protein bands were visualized using an enhanced chemiluminescence system (Bio‐Rad, Hercules, CA, USA) and quantified by ImageJ software (National Institutes of Health, USA).

### 5.20 Detection of Autophagic Flux (DALGreen and DAPRed)

To evaluate the impact of SCFAs on autophagic flux, we performed autolysosome and autophagosome staining as previously described [23]. Briefly, NRCMs were coincubated with 1.0 µmol/L DALGreen and 0.2 µmol/L DAPRed (Dojindo, Japan) for 30 min at 37°C. Following HG exposure and treatment with butyrate or propionate for 24 h, cells were washed with PBS and visualized using an Olympus confocal microscope (Japan). The formation of acidic autolysosomes was quantified by measuring the fluorescence intensity and puncta counts per cell. To ensure comparability, identical imaging parameters were maintained across all groups, and data were analyzed by investigators blinded to the experimental groups.

### 5.21 Statistical Analysis

Statistical analysis was performed using GraphPad Prism v9.0 (GraphPad Software, San Diego, CA, USA). Data are presented as mean ± standard error of the mean (SEM). The normality of data distribution was assessed using the Shapiro–Wilk test. For normally distributed data, statistical differences between two groups were determined by unpaired Student's *t*‐test, while comparisons among multiple groups were analyzed using one‐way ANOVA followed by Tukey's posthoc test. For non‐normally distributed data, the Mann–Whitney *U*‐test or Kruskal–Wallis test followed by Dunn's multiple comparison test was utilized.

A value of *p* < 0.05 was considered statistically significant (*p < 0.05, **p < 0.01, ***p < 0.001, ****p < 0.0001.).

## Author Contributions

Yongjun Wang and Shi Tai: conceptualization and methodology. Shi Tai, Zhiyi Yin, Yuying Zhou, and Liyao Fu: software. Yuying Zhou, Zhiyi Yin, and Liyao Fu: validation. Shi Tai, Zhiyi Yin, Yuying Zhou, Hejun Jiang, and Liyao Fu: formal analysis. Yongjun Wang, Shi Tai, Zhiyi Yin, Yuying Zhou, Xunxi Deng, Ying Zhou, and Liyao Fu: investigation. Yongjun Wang and Shi Tai: resources. Yuying Zhou, Zhiyi Yin, and Shi Tai: data curation. Yuying Zhou and Zhiyi Yin: Writing – original draft. Yongjun Wang, Shi Tai, and Liyao Fu: Writing – review and editing. Yuying Zhou, Zhiyi Yin, Xunxi Deng, Hejun Jiang, Ying Zhou, and Shi Tai: visualization. Yongjun Wang, Shi Tai, and Liyao Fu: supervision. Yongjun Wang, Shi Tai, and Liyao Fu: project administration. Shi Tai and Liyao Fu: funding acquisition. All authors have read and approved the final manuscript.

## Funding

This research was supported by the National Natural Science Foundation of China (82271601) and the Natural Science Foundation of Hunan Province (2023JJ30780 and 2022JJ40683).

## Conflicts of Interest

The authors declare no conflicts of interest.

## Ethics Statement

All experiments involving animals were conducted according to the ethical policies and procedures approved by the Ethics Committee of the Second Xiangya Hospital of Central South University, Changsha, China (Approval no. 2022400) and in line with the Guide for the Care and Use of Laboratory Animals, NIH publication, 8th edition, 2011.

## Supporting information




**Supporting File: 1** mco270910‐supp‐0001‐SuppMat.docx

## Data Availability

The RNA‐seq data generated in this study have been deposited in the Genome Sequence Archive (GSA) at the National Genomics Data Center (NGDC), China National Center for Bioinformation (CNCB), under the accession number PRJCA063102. All other data supporting the findings of this study are available within the article and its supplementary materials, or from the corresponding author upon reasonable request.
