# Supplementary material for: Time‐Restricted Feeding Prevents Cardiac Aging by Entraining Gut Microbiota Clock and Promoting Diurnal Rhythm Autophagy in Prediabetic Mice
Source: MedComm (2020). 2026 Aug 9;7(8):e70910. doi: 10.1002/mco2.70910 (PMC13454559; doi:10.1002/mco2.70910)
Supplement: Supplementary file 1 [file MCO2-7-e70910-s001.docx]

**Time-restricted feeding prevents cardiac aging by entraining gut microbiota clock and promoting diurnal rhythm autophagy in pre-diabetic mice**

Yuying Zhou^1#^, Zhiyi Yin^1,2#^, Xunxi Deng^1,2^, Hejun Jiang^1^, Ying Zhou^1,2^, Yongjun Wang^2^**^*^**, Liyao Fu^1,2^**^*^**, Shi Tai^1^**^*^**

1. Department of Cardiology, The Second Xiangya Hospital of Central South University, Changsha 410011, China

2. Department of Blood Transfusion, The Second Xiangya Hospital of Central South University, Changsha, China

^#^These authors contributed equally to this work.

^*^Correspondence: Shi Tai ([taishi2017@csu.edu.cn](mailto:taishi2017@csu.edu.cn));

Liyao Fu ([fuliyao2017@csu.edu.cn](mailto:fuliyao2017@csu.edu.cn));

Yongjun Wang ([wangyongjun@csu.edu.cn](mailto:wangyongjun@csu.edu.cn)).

**Running Title:** Time-restricted feeding prevents cardiac aging

**Supplementary Figure 1**

(A) Schematic representation of the feeding regimens and group color-coding. The time-restricted feeding (TRF) group was restricted to a 10-hour feeding window from 22:00 to 08:00 (ZT14 to ZT24/ZT0), aligning with the natural nocturnal activity and feeding rhythm of mice. During the remaining non-feeding period (08:00 to 22:00, ZT0 to ZT14), the TRF group was subjected to fasting. The ad libitum feeding (ALF) group had unrestricted access to food throughout the 24-hour cycle.

(B) Representative photographs depicting the gross body morphology and physical appearance of mice across groups.

(C-D) Assessment of glucose homeostasis via intraperitoneal glucose tolerance test (IPGTT) curves (C) and the corresponding area under the curve (AUC) calculation (D) (n = 26–37). In the glucose excursion curves (D), the top-tier curves represent the diabetic groups, the middle-tier curves represent the pre-diabetic groups, and the bottom curve represents the normal control group.

(E-F) Dynamic changes in body weight and blood glucose throughout the experimental period (n = 11–20).

(G) Allmeters, water consumption and an index of fine activity from metabolic cages recordings (n=3).

(H-I) Measurements of the dietary intake per cage of mice in five groups (n=3-4).

Data are presented as mean ± SEM. *P<0.05,**P<0.01,***P<0.001,****P<0.0001.

Consistent color-coding is used to distinguish the experimental groups: dark blue for N-ALF, orange for P-ALF, light green for P-TRF, brown for D-ALF, and dark red for D-TRF.

**Supplementary Figure 2**

(A) Quantitative echocardiographic parameters: LV internal diameter in systole (LVIDs) and diastole (LVIDd), Tei index, LV anterior wall thickness in diastole (LVAWd), left ventricular anterior wall thickness in systole (LVAWs), left ventricular posterior wall thickness in systole (LVPWs) and diastole (LVPWd) (n=4-5).

(B-D) Serum concentrations of SASP factors, including interleukin-6 (IL-6, B), interleukin-8 (IL-8, C), and transforming growth factor-beta (TGF-β, D) assessed by ELISA (n = 5–7).

(E) Qualitative and quantitative assessment of mitochondrial morphology. Mitochondrial scoring criteria based on cristae integrity: 4 (cristae >80%), 3 (60%–80%), 2 (30%–60%), 1 (15%–30%), and 0 (<15%).

Data are presented as mean ± SEM. *P<0.05,**P<0.01,***P<0.001,****P<0.0001.

Consistent color-coding is used to distinguish the experimental groups: dark blue for N-ALF, orange for P-ALF, light green for P-TRF, brown for D-ALF, and dark red for D-TRF.

**Supplementary Figure 3**

(A) Volcano plot showing the differentially expressed genes (DEGs) between P-T and P-A mice. Each blue dot denotes a gene with adjusted p < 0.05 and log2(fold change) < -1. Each red dot denotes a gene with adjusted p < 0.05 and log2(fold change) >1.

(B) Chord plot showing the GO pathway enrichment analysis of the top genes in hearts between P-T and P-A mice.

(C)Volcano plot showing the differentially expressed genes (DEGs) between D-T and D-A mice. Each blue dot denotes a gene with adjusted p < 0.05 and log2(fold change) < -1. Each red dot denotes a gene with adjusted p < 0.05 and log2(fold change) >1.

(D) Chord plot showing the GO pathway enrichment analysis of the top genes in hearts between D-T and D-A mice.

(E) Bubble plot showing the Kyoto Encyclopedia of Genes and Genomes (KEGG) pathway enrichment analysis of the top 10 pathways in hearts between D-T and D-A mice.

(F) Chord plot showing the KEGG pathway enrichment analysis of the top genes in hearts between D-T and D-A mice.

**Supplementary Figure 4**

(A,C,D) Representative immunofluorescence (IF) images and quantitative analysis of p62/PINK1 oscillations in pre-diabetic mice hearts. (scale bar = 20 μm, n = 3).

(B,E,F) Representative IF staining and quantitative analysis of p62/PINK1 oscillations in diabetic mice hearts (scale bar = 20 μm, n = 3).

Sections were stained for p62 or PINK1 (green) and counterstained with cardiac Troponin T (cTnT, red) to demark cardiomyocytes. Nuclei were stained with DAPI (blue).

Data are presented as mean ± SEM. *P<0.05,**P<0.01,***P<0.001,****P<0.0001.

ZT, Zeitgeber Time; ZT0 indicates the onset of the light phase (08:00), and ZT12 indicates the onset of the dark phase (20:00).

**Supplementary Figure 5**

(A-B) Representative immunoblot images (A) and measurements of the expression level (B) for p62, PINK1 in P-ALF mice. GAPDH was utilized for normalization (n=3-4).

(C-D) Representative immunoblot images (C) and measurements of the expression level (D) for p62, PINK1 in D-ALF mice. GAPDH was utilized for normalization (n=3-4).

(E-F) Representative immunoblot images (E) and measurements of the expression level (F) for p62, PINK1 in N-ALF mice. GAPDH was utilized for normalization (n=3-4).

Data are presented as mean ± SEM. *P<0.05,**P<0.01,***P<0.001,****P<0.0001.

**Supplementary Figure 6**

(A) Box plot showing the Shannon index between P-TRF and P-ALF mice (left), the shannon index between D-TRF and D-ALF mice (right).

(B) Bar plot showing the taxonomic composition of intestinal contents in P-TRF and P-ALF mice (left), the taxonomic composition every 6 hours in P-TRF mice (right).

(C) Bar plot showing the taxonomic composition of intestinal contents in D-TRF and D-ALF mice (left), the taxonomic composition every 6 hours in D-TRF mice (right).

**Supplementary Figure 7**

(A-B) Analysis of specific microbial taxa (*Enterobacteriaceae*, *Alloprevotella*, *Roseburia*, and *Blautia*) in pre-diabetic mice, including their relative abundance (n = 14–17) and diurnal oscillation patterns across four ZT points (n = 3–4).

(C-D) Analysis of specific microbial taxa in diabetic mice, including their relative abundance (n = 18–23) and diurnal oscillation patterns across four ZT points (n = 4–7).

Data are presented as mean ± SEM. *P<0.05,**P<0.01,***P<0.001,****P<0.0001.

ZT, Zeitgeber Time; ZT0 indicates the onset of the light phase (08:00), and ZT12 indicates the onset of the dark phase (20:00).

**Supplementary Figure 8**

(A) Representative images of senescence-associated β-galactosidase (SA-β-gal) staining and corresponding quantification in NRCMs (n = 3). Scale bar = 100 μm.

(B-D) Representative immunoblotting (IB) images and quantitative analysis of autophagy markers p62 and LC3-II/I in HG-induced senescent NRCMs following propionate treatment (n = 3).

(E-J) Assessment of autophagic flux using Bafilomycin A1 (Baf A1) in HG-induced senescent NRCMs. NRCMs were treated with propionate or butyrate (1 mM) for 24 h, with Baf A1 (100 nM) added during the final 2 h of incubation. Representative IB images and quantitative analysis of p62 and LC3-II/I are shown (n = 3).

GAPDH and β-actin served as loading controls for the butyrate and propionate experimental groups, respectively.

Data are presented as mean ± SEM. *P<0.05,**P<0.01,***P<0.001,****P<0.0001.

**Supplementary Figure 9**

(A) The abundance of Lactobacillus(n=17-18), Bifidobacterium (n=17-18), Lachnospiraceae(n=15-17) and Coprostanoligenes(n=6) in P-TRF and P-ALF mice.

(B) The abundance of Lactobacillus(n=22), Bifidobacterium (n=15-23), Lachnospiraceae(n=18-19) and Coprostanoligenes(n=7-12) in P-TRF and P-ALF mice.

(C) Representative immunofluorescence staining of cTnT (green) in NRCMs, showing the purity of isolated NRCMs (Scale bar = 50μm).

Data are presented as mean ± SEM. *P<0.05,**P<0.01,***P<0.001,****P<0.0001.
